# Using Expansion Microscopy to Visualize and Characterize the Morphology of Mitochondrial Cristae

**DOI:** 10.3389/fcell.2020.00617

**Published:** 2020-07-15

**Authors:** Tobias C. Kunz, Ralph Götz, Shiqiang Gao, Markus Sauer, Vera Kozjak-Pavlovic

**Affiliations:** ^1^Department of Microbiology, Julius-Maximilians-Universität Würzburg, Würzburg, Germany; ^2^Department of Biotechnology and Biophysics, Julius-Maximilians-Universität Würzburg, Würzburg, Germany; ^3^Department of Botany I, Julius-Maximilans-Universität Würzburg, Würzburg, Germany

**Keywords:** Expansion microscopy, mitochondria, cristae, structured illumination microscope, ultrastructure

## Abstract

Mitochondria are double membrane bound organelles indispensable for biological processes such as apoptosis, cell signaling, and the production of many important metabolites, which includes ATP that is generated during the process known as oxidative phosphorylation (OXPHOS). The inner membrane contains folds called cristae, which increase the membrane surface and thus the amount of membrane-bound proteins necessary for the OXPHOS. These folds have been of great interest not only because of their importance for energy conversion, but also because changes in morphology have been linked to a broad range of diseases from cancer, diabetes, neurodegenerative diseases, to aging and infection. With a distance between opposing cristae membranes often below 100 nm, conventional fluorescence imaging cannot provide a resolution sufficient for resolving these structures. For this reason, various highly specialized super-resolution methods including *d*STORM, PALM, STED, and SIM have been applied for cristae visualization. Expansion Microscopy (ExM) offers the possibility to perform super-resolution microscopy on conventional confocal microscopes by embedding the sample into a swellable hydrogel that is isotropically expanded by a factor of 4–4.5, improving the resolution to 60–70 nm on conventional confocal microscopes, which can be further increased to ∼ 30 nm laterally using SIM. Here, we demonstrate that the expression of the mitochondrial creatine kinase MtCK linked to marker protein GFP (MtCK-GFP), which localizes to the space between the outer and the inner mitochondrial membrane, can be used as a cristae marker. Applying ExM on mitochondria labeled with this construct enables visualization of morphological changes of cristae and localization studies of mitochondrial proteins relative to cristae without the need for specialized setups. For the first time we present the combination of specific mitochondrial intermembrane space labeling and ExM as a tool for studying internal structure of mitochondria.

## Introduction

Super-resolution imaging has revolutionized fluorescence imaging by its capability to bypass the resolution limit of optical microscopy as defined by Abbe (1873). The most common methods, stimulated emission depletion (STED) microscopy (Hell, 2007), photoactivated localization microscopy (PALM) (Betzig et al., 2006) and (*direct)* stochastic optical reconstruction microscopy (*d)*STORM (Heilemann et al., 2008), were applied to countless biological specimens and can provide new insights into cellular structures and tissue in 2D and 3D (Xu et al., 2013; Michie et al., 2017).

However, super-resolution techniques enabling a resolution <100 nm require specialized setups and expert knowledge to avoid artifacts (Burgert et al., 2015). Expansion microscopy (ExM) (Chen et al., 2015) in contrast avoids this need by physical expansion of the sample after embedding into a polyacrylamide gel. Various protocols have been established using either a digestion or a denaturation step with numerous staining and linking protocols (Chozinski et al., 2016; Ku et al., 2016; Tillberg et al., 2016; Gambarotto et al., 2019; Götz et al., 2020). With an expansion of ∼4–4.5 times, ExM empowers scientists to resolve structures with a lateral resolution of ∼60–70 nm on a confocal microscope and in combination with structured illumination microscopy (SIM) (Gustafsson, 2000) of even ∼30 nm, approaching the resolution of other conventional super-resolution methods (Wang et al., 2018).

Many important biochemical processes take place in mitochondria, from the determination of cell fate by apoptosis induction, the citric acid cycle, and the production of metabolites, to the energy conversion *via* cell respiration. The latter is taking place at the mitochondrial inner membrane, which increases its surface by folding into cristae. The inner mitochondrial membrane can therefore be divided into the regions of the cristae membrane, which projects into the matrix, and the inner boundary membrane, which is found opposite to the outer mitochondrial membrane. Two regions meet at the so-called cristae junction (Frey and Mannella, 2000). Changes in morphology of cristae have been associated with aging, numerous diseases, such as cancer, diabetes, several neurodegenerative diseases or types of neuro- and myopathies, and infection (Kozjak-Pavlovic et al., 2009; Bohnert et al., 2015; Cogliati et al., 2016; Kondadi et al., 2019). Thus, the possibility to investigate cristae morphology and the localization of mitochondrial proteins is of broad interest. Up to now, most light microscopy approaches have been performed using STED (Schmidt et al., 2009; Stephan et al., 2019; Wang et al., 2019) or Airyscan microscopy (Wolf et al., 2019). Although very successful in cristae visualization, the limitation is the restricted availability of super-resolution microscopes in standard cell biology laboratories as tools for investigating the mitochondrial ultrastructure.

Here, we report that ExM offers the possibility to image mitochondrial cristae on a classical confocal microscope and to localize mitochondrial proteins with an estimated lateral resolution of ∼30 nm in combination with SIM. We used green fluorescent protein (GFP)-labeled mitochondrial intermembrane space protein, mitochondrial creatine kinase (MtCK-GFP), as a cristae marker, and antibodies against mitochondrial matrix and cristae-associated proteins. As an example of the applicability of this technique, using the combined resolution power of ExM and SIM we demonstrate that the mitochondrial transcription factor TFAM associates with cristae, and we observe changes in mitochondrial morphology after membrane potential dissipation by CCCP or knockdown of the member of the mitochondrial intermembrane space bridging complex (MIB), Sam50.

## Materials and Methods

### Cell Culture

Human HeLa229 cells (ATCC CCL-2.1tm) and Sam50 knockdown cells *sam50kd-2* (Kozjak-Pavlovic et al., 2007) were cultured in 10% (v/v) heat inactivated FBS (Sigma-Aldrich, St. Louis, MO, United States) RPMI1640 + GlutaMAXtm medium (Gibco, Thermo Fisher Scientific, Waltham, MA, United States). The cells were grown in a humidified atmosphere containing 5% (v/v) CO_2_ at 37°C. For the induction of the shRNA-mediated knockdown of Sam50 cells were treated with 1 μg/ml doxycycline for 72 h prior seeding.

### Transfection

MtcK gene was amplified from HeLa cDNA and cloned into the pCDNA3 vector (Thermo Fisher Scientific, Waltham, MA, United States) where previously the GFP sequence was introduced, enabling C-terminal fusion and tagging. HeLa cells were transfected using Viromer^®^ RED (230155; Biozym, Oldendorf, Germany) according to manufacturer’s instructions.

### Antibody Conjugation

Following buffer exchange to 100 mM NaHCO_3_ with 0.5 ml 7 kDa Spin Desalting Columns (89882; Thermo Fisher Scientific, Waltham, MA, United States), the anti-TFAM (TA332462, rabbit; Origene, Rockville, United States) antibody was incubated in 5 molar excess of NHS-Alexa Fluor 546 (A20002; Thermo Fisher Scientific, Waltham, MA, United States) or NHS-ATTO 643 (AD 643-31; ATTO-TEC; Siegen, Germany), for 3 h at RT. After conjugation, the unreacted dye was filtered from the antibody using 0.5 ml 7 kDa Spin Desalting Columns and the buffer was exchanged to 0.02% NaN_3_ dissolved in PBS. The degree of labeling (DOL) was determined by the absorption of the antibody-dye with a UV-vis spectrophotometer (Jasco V-650). The labeled antibody was stored at 4°C.

### Immunostaining

Twenty four hours after transfection, the cells were washed with 1xPBS and fixed with 4% PFA for 30 min at RT. Afterward the cells were washed with 1xPBS, permeabilized for 15 min in 0.2% Triton-X100 and then blocked for 1 h in 2% FCS. Upon blocking, the cells were incubated for 1 h in primary antibody in a humidified chamber. We used the following primary antibodies: α-PRX3 (TA322472, rabbit; Origene, Rockville, United States), α-Mitofilin (ab48139, rabbit; Abcam, Cambridge, United Kingdom), α-TFAM (TA332462, rabbit; Origene, Rockville, United States), α-GFP (ab1218, mouse; Abcam, Cambridge, United Kingdom or SP3005P, rabbit; Origene, Rockville, United States), α-TOM20 (sc-17764, mouse; Santa Cruz, Dallas, United States) and α-TIM44 (612582, mouse; BD Biosciences, Franklin Lakes, United States). All primary antibodies were used in a dilution of 1:100 except of α-Tom20 (1:25) and α-Tim44 (1:50). After incubation with the primary antibody, the cells were incubated with the secondary antibody, Alexa-488 (A11017, dilution 1:200, goat anti-mouse Alexa 488, Thermo Fisher Scientific, Waltham, MA, United States), Alexa-488 (A11070, dilution 1:200, goat anti-rabbit Alexa 488, Thermo Fisher Scientific, Waltham, MA, United States), self-conjugated NHS-ATTO643 (AD 643-31; ATTO-TEC; Siegen, Germany) and ATTO647N (610-156-121S, dilution 1:200, goat anti-rabbit ATTO 647N, Rockland Immunochemicals, Pottstown, United States). For 3 color images, the cells were then incubated with anti TFAM-Alexa 546 antibody (TA332462 self-conjugated or sc-166965; Santa Cruz Biotechnology, Dallas, United States).

### Expansion Microscopy

Expansion Microscopy was performed as described previously (Chozinski et al., 2016; Kunz et al., 2019). Stained samples were incubated for 10 min in 0.25% glutaraldehyde, washed and gelated with a monomer solution containing 8.625% sodium acrylate (408220, Sigma-Aldrich, St. Louis, MO, United States), 2.5% acrylamide (A9926, Sigma-Aldrich, St. Louis, MO, United States), 0.15% N,N′-methylenbisacrylamide (A9926, Sigma-Aldrich, St. Louis, MO, United States), 2 M NaCl (S5886, Sigma-Aldrich, St. Louis, MO, United States), 1x PBS, 0.2% ammonium persulfate (APS, A3678; Sigma-Aldrich, St. Louis, MO, United States) and tetramethylethylenediamine (TEMED, T7024; Sigma-Aldrich, St. Louis, MO, United States). Note that TEMED and KPS were added just prior to gelation and gelation was performed for 1 h at RT in humidified gelation chambers. The gelated samples were digested in the appropriate proteinase-containing buffer [50 mM Tris pH 8.0, 1 mM EDTA (ED2P, Sigma-Aldrich, St. Louis, MO, United States), 0.5% Triton X-100 (28314, Thermo Fisher Scientific, Waltham, MA, United States) and 0.8 M guanidine HCl (50933, Sigma-Aldrich, St. Louis, MO, United States)] for 30 min and consequently expanded in ddH_2_O. The expansion factor was determined by the gel size prior and after expansion. Expanded gels were stored at 4°C in ddH_2_O until use and immobilized on PDL coated imaging chambers (734–2055, Thermo Fischer Scientific, Waltham, MA, United States) for imaging.

### Microscopes

Imaging of the unexpanded and expanded specimen was performed on a confocal system [Zeiss LSM700 5 mW red laser (637 nm) and a 10 mW blue laser (488 nm)] and on a structured illumination microscope (Zeiss Elyra S.1 SIM). In both cases a water objective (C-Apochromat, 63 × 1.2 NA, Zeiss, 441777-9970) was used and images were processed with Imaris 8.4.1 and FIJI 1.51n (Schindelin et al., 2012).

*d*STORM imaging was carried out on a home-built setup using an inverted widefield microscope (Olympus IX-71) equipped with an oil immersion objective (Olympus APON 60xO TIRF, NA 1.49) and an excitation laser of the wavelength 639 nm (Genesis MX639-1000, Coherent). The excitation beam was separated from the emitted fluorescence via a dichroic mirror (ZT405/514/635rpc, Chroma) and the emission was additionally filtered by an emission filter [Brightline HC 679/41 (Semrock)] in front of the EMCCD-camera (iXon Ultra 897, Andor). Prior to imaging, a switching buffer containing 100 mM ß-mercaptoethylamine pH 7.4 was added and 15 000 Frames at 50 Hz were recorded using laser densities of ∼ 7 kW/μm^2^. The super resolved images were reconstructed using the software rapidSTORM 3.3 (Wolter et al., 2012).

## Results

**FIGURE 1:**
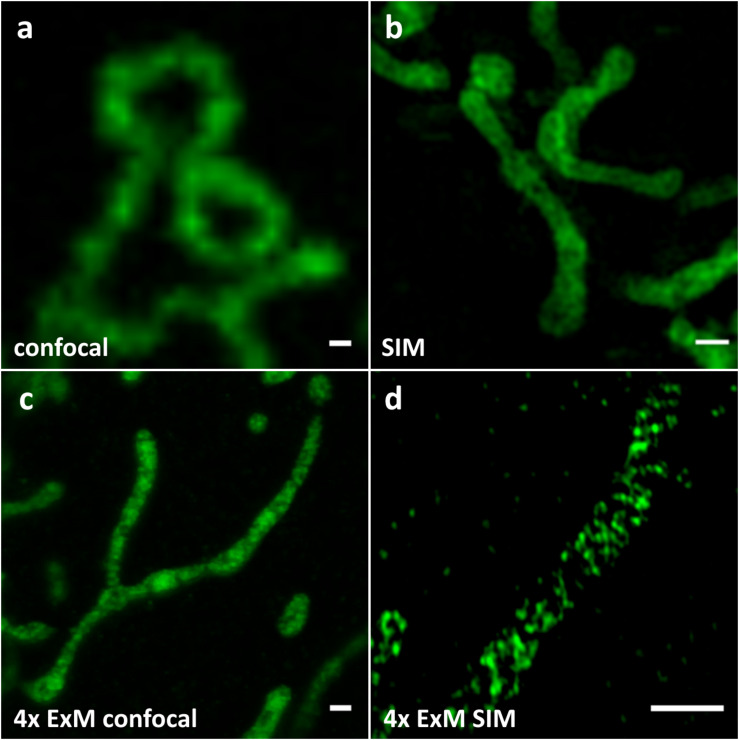
Comparison of **(a)** confocal, **(b)** SIM, **(c)** 4x ExM confocal **(d)** 4x ExM SIM images of Hela229 cells, transfected for 24 h with MtCK-GFP (green) and immunolabeled for GFP to visualize mitochondrial cristae. Scale bars, **(a,b)** 0.5 μm and **(c,d)** 2 μm.

Our first goal was to find a marker protein localizing to mitochondrial cristae, which can be expressed and correctly targeted when fused to a fluorescent protein without affecting mitochondrial function or morphology. After testing several candidates, we chose the MtCK, an enzyme providing a temporal and spatial energy buffer to maintain cellular energy homeostasis by creating phosphocreatine using mitochondrial ATP (Schlattner et al., 2006). This protein localizes to mitochondrial intermembrane space, and after the transfection of HeLa229 cells and expression of its carboxy-terminus GFP-labeled version MtCK-GFP, we observed no effect on cell viability or mitochondrial length and distribution (not shown). The transfected Hela229 cells expressing MtCK-GFP were decorated with anti-GFP antibody prior to expansion, which we performed using the Chozinski protocol (Chozinski et al., 2016; Figure 1). Non-expanded and expanded cells were imaged by confocal fluorescence microscope (Figures 1a,c) and SIM (Figures 1b,d) and compared with *d*STORM images of unexpanded cells (Supplementary Figure S1). Confocal imaging of expanded samples (Figure 1c) was comparable in resolution with the SIM of unexpanded cells (Figure 1b), whereas the combination of 4x ExM and SIM provided the best resolution of internal mitochondrial structure (Figure 1d).

**FIGURE 2:**
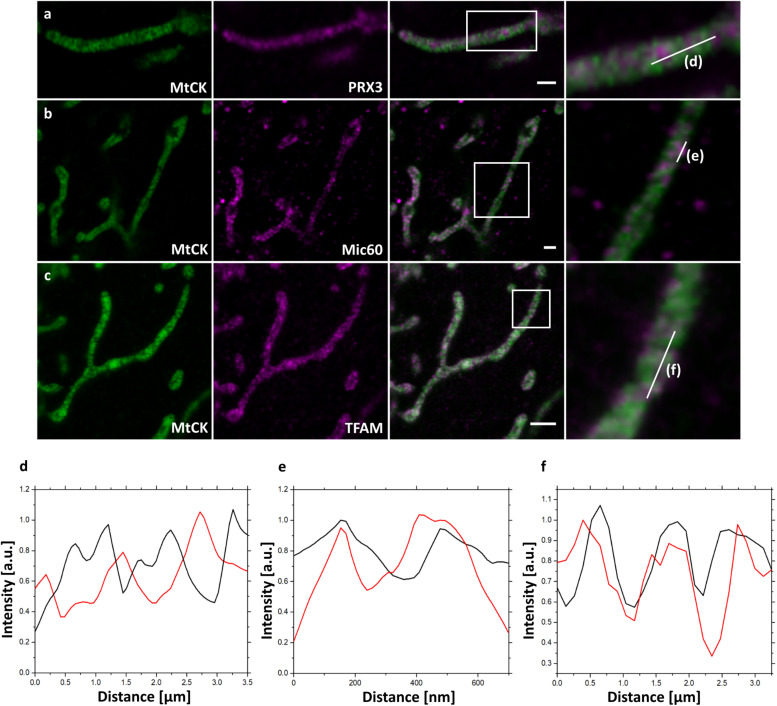
Confocal fluorescence images of 4x expanded HeLa229 cells transfected for 24 h with MtCK-GFP (green) to visualize cristae, immunolabeled for GFP and **(a)** PRX3, **(b)** Mic60/Mitofilin or **(c)** TFAM (shown in magenta). Plot profiling of the fluorescence of MtCK (black) relative to **(d)** PRX3, **(e)** Mic60/Mitofilin and **(f)** TFAM (red). 4x ExM can resolve if mitochondrial proteins localize at mitochondrial cristae as demonstrated for Mic60 and TFAM. White boxes highlight the zoomed regions shown at the panel to the right. Scale bars, 2 μm.

In addition to the GFP antibody labeling, MtCK-GFP-expressing cells were next decorated with antibodies against several mitochondrial proteins before expansion and analyzed by confocal fluorescence microscope after 4x ExM (Figure 2). We chose peroxiredoxin 3 (PRX3), a mitochondrial thioredoxin-dependent hydroperoxidase present in mitochondrial matrix (Donsante, 2017), to assess the efficiency of MtCK-GFP to depict cristae with its uniform distribution through the intermembrane space (Figure 2a). This was indeed the case, since the signals for MtCK-GFP (green) and PRX3 (magenta) alternated and did not overlap, showing that 4x ExM enables the differentiation between the cristae and the matrix (Figure 2d). Next, we used the combined staining for GFP and Mic60/Mitofilin. The latter protein is one of the central components of the inner membrane mitochondrial cristae organizing system (MICOS), which together with the sorting and assembly machinery (SAM) in the outer mitochondrial membrane forms the mitochondrial intermembrane space bridging complex (MIB), a large complex necessary for the maintenance of mitochondrial cristae morphology, cristae junctions, inner membrane architecture, and formation of contact sites between two mitochondrial membranes (John et al., 2005; Ott et al., 2015; Kozjak-Pavlovic, 2017). As we expected, we were able to localize Mic60/Mitofilin signals closer to the mitochondrial surface (Figure 2b) and overlapping with the signals of MtCK-GFP (Figure 2e). Finally, we used ExM to determine the localization of mitochondrial transcription factor A (TFAM) in relation to cristae. TFAM associates with mitochondrial DNA (mtDNA) nucleoids in the matrix, but a connection exists between cristae formation and mitochondrial nucleoid organization (Li et al., 2016). Contrary to PRX3, TFAM is heterogeneously distributed in the matrix localized to punctate structures, which we observed overlapping with the MtCK-GFP signal (Figures 2c,f). This indicates that TFAM and mitochondrial nucleoids are found in the vicinity of cristae.

**FIGURE 3:**
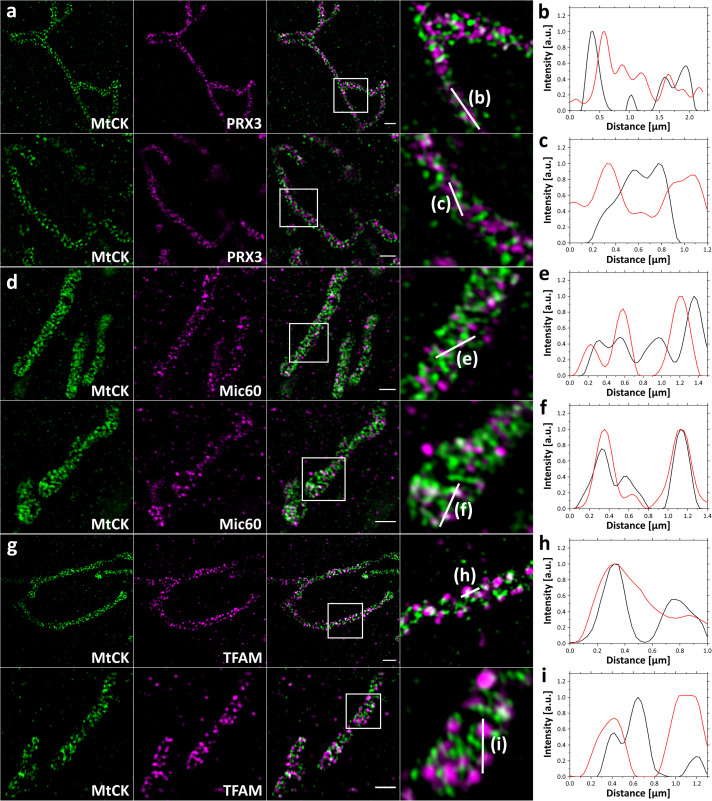
4x SIM-ExM of MtCK and mitochondrial marker proteins. HeLa229 cells were transfected with MtCK-GFP for 24 h, fixed, immunolabeled for GFP and PRX3 **(a)**, Mic60 **(d)** or TFAM **(g)** and expanded. MtCK-GFP is depicted in green, while PRX3, Mic60 and TFAM are shown in magenta. Within the overlay the zoomed region (white box) is indicated with the corresponding region of the line profile **(b,c,e,f,h,i)**. PRX3 alternates with MtCK while Mic60 and TFAM partially colocalize with MtCK. Scale bars, 2 μm.

This finding was supported by SIM imaging of 4x expanded cells. The improved lateral resolution of ∼30 nm allowed us to visualize that here again mitochondrial matrix protein PRX3 localized between individual cristae (Figure 3a), and the inner membrane protein Mic60 localized in the vicinity of the cristae junctions (Figure 3d). We also observed that most of the TFAM signal is found at the mitochondrial periphery, colocalizing with the MtCK-GFP signal, which indicates a possible association of TFAM and mtDNA nucleoids with cristae junction (Figure 3g). These observations are further confirmed by the corresponding plot profiles (Figures 3b,c,e,f,h,i).

To demonstrate potential applications of ExM for mitochondrial studies we performed additional experiments and co-stained MtCK-GFP-labeled mitochondria with TOM20, a component of the translocase of the outer membrane (TOM) complex, or TIM44, a component of the translocase of the inner membrane (TIM) complex, antibodies. In both cases ExM enabled an efficient discrimination of MtCK-GFP cristae signal compared to the outer membrane or the inner boundary membrane where TOM and TIM complexes are found, respectively (Supplementary Figure S2).

**FIGURE 4:**
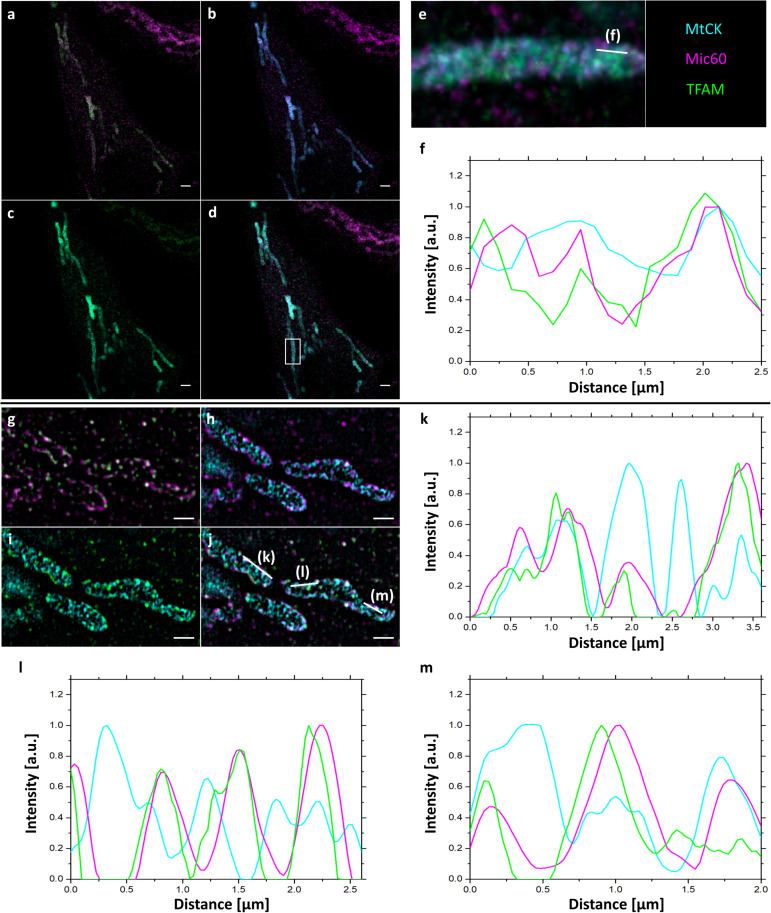
4x ExM of 3-color confocal **(a–e)** and SIM **(g–j)** images of the localization of mitochondrial proteins relative to mitochondrial cristae. Hela229 cells were transfected with MtCK-GFP for 24 h, fixed, immunolabeled for GFP, Mitofilin and TFAM and expanded. **(a,g)** Mitofilin (magenta) and TFAM (green), **(b,h)** MtCK (cyan) and Mitofilin (magenta), **(c,i)** MtCK (cyan), and TFAM (green), **(d,j)** merge, **(e)** zoom from **(d)** (white box) with line indicating plot profiling shown in **(f)**. **(k–m)** plot profiling line shown in **(j)**. Scale bars, 5 μm for confocal images, 2 μm for SIM images.

Next, we performed 3-color confocal (Figures 4a–e) and SIM (Figures 4g–m and Supplementary Figure S3) imaging of cristae, Mic60/Mitofilin and TFAM in expanded samples to better compare mitochondrial proteins and their localizations. The signals for all three proteins largely overlapped, but interestingly, the colocalization of Mic60/Mitofilin and TFAM was somewhat stronger than observed for Mic60/Mitofilin and MtCK-GFP (Figures 4f,k–m and Supplementary Figure S3). This result confirms our previous observation that TFAM might localize in the vicinity of cristae junctions, which are marked by the presence of Mic60/Mitofilin.

**FIGURE 5:**
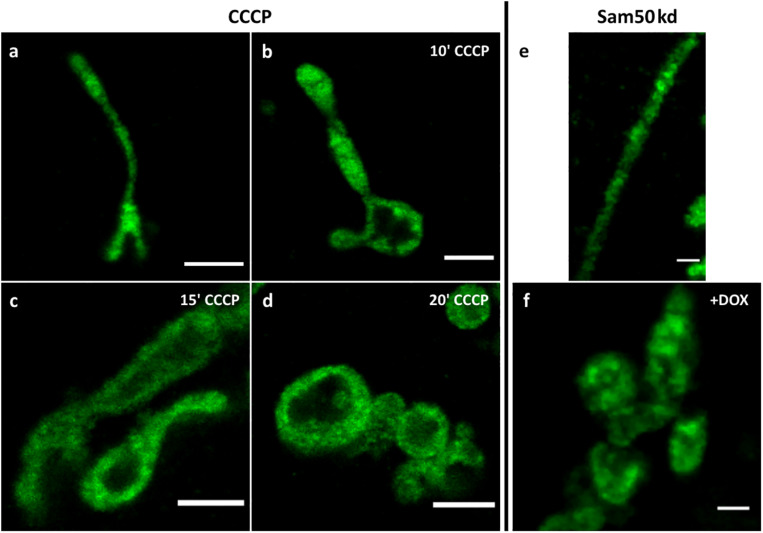
Confocal images of 4x expanded HeLa229 cells **(a–d)**, transfected with MtCK-GFP (green), treated with 1 μM CCCP for 10 **(b)**, 15 **(c)** or 20 **(d)** minutes. **(e,f)** Sam50 knockdown cells (sam50kd-2) were treated for 72 h with 1 μg/ml doxycycline to induce Sam50 knockdown, transfected with MtCK-GFP (green) for 24 h and analyzed by ExM on a confocal microscopy **(f)**. The respective control sample without induced Sam50 knockdown is shown in **(e)**. Scale bars, 2 μm.

To demonstrate the usefulness of 4x ExM for determining the morphology of mitochondrial cristae, we treated cells transfected with MtCK-GFP with 1 μM CCCP, a strong uncoupling agent that can abruptly depolarize the membrane potential (Park et al., 1997). Cells incubated with CCCP exhibited rounding and swelling of mitochondria in a time-dependent manner, which was successfully visualized using MtCK-GFP and ExM (Figures 5a–d). Additional 4x ExM SIM shows that swollen mitochondria still displayed cristae, although their density was reduced in comparison to non-treated mitochondria (Supplementary Figure S4). Furthermore, we transfected HeLa cells where the knockdown of Sam50 could be induced by doxycycline (Dox)-mediated shRNA expression (*sam50kd-2*) with MtCK-GFP. Sam50 is a component of the SAM and MIB complex and, in addition to its function in the sorting of proteins with complicated topology, such as β-barrel proteins (Kozjak et al., 2003), it is also important for the maintenance of cristae integrity. The enlargement of mitochondria and loss of cristae structure, that has already been visualized by transmission electron microscopy (Ott et al., 2015), was also visible after ExM and analysis of the samples by confocal microscopy (Figures 5e,f).

## Discussion

In this study we could demonstrate the visualization of individual cristae on a conventional confocal microscope by applying ExM. This approximately fourfold expansion resulting in a resolution of ∼60 nm (Chen et al., 2015) appears to be a promising tool for mitochondrial research, especially for cristae, folds of the inner membrane, with a distance often below 100 nm (Jakobs and Wurm, 2014). Imaging of cristae, however, has always been challenging due to the limited resolution in light microscopy. Hence, first successful results could be performed only with the advent of super resolution microscopy by applying SMLM (Shim et al., 2012), STED (Schmidt et al., 2009) or SIM (Huang et al., 2018) approaches. While SMLM and STED have the limitation of highly specialized setups and intensive training, SIM has the drawback of an only to ∼ 100 nm limited resolution. Recently, studies showed great improvements in live-cell imaging using STED or Airyscan microscopy. These methods were used in combination with fluorescent dyes or inner membrane markers for analysis of the mitochondrial ultrastructure (Stephan et al., 2019; Wang et al., 2019), measurement of the membrane potential of individual cristae (Wolf et al., 2019) or even cristae dynamics (Kondadi et al., 2020). Although these live-cell imaging methods enabled following of cristae dynamics in real time, they also exhibited very fast damaging of mitochondrial vitality and are also not suitable for protein localization studies.

Contrary to those already established cristae markers COX8A (Stephan et al., 2019) or ATP5I (Kondadi et al., 2020), we assume that we do not interfere with the naïve ATP production cycle using the mitochondrial creatine kinase MtCK as a cristae marker. Other fluorescent dyes like MitoPB Yellow (Wang et al., 2019) or MitoTracker for labeling cristae were unfortunately not compatible with ExM due to the lack of a primary amine, making MtCK a preferred possibility to label cristae.

Our approach of expanding exclusively fixed samples offers the possibility of immunolabeling and thus enables studies of protein localization relative to mitochondrial cristae with an estimated resolution of 60 nm. By additionally applying SIM the resolution is improved to ∼30 nm (Figures 3, 4g–j and Supplementary Figure S3), approaching super-resolution methods like *d*STORM (Supplementary Figure S1) and STED. Moreover, ExM also offers the possibility of super-resolved imaging of 3 or even 4 colors of the whole cell, a severe obstacle in super-resolution microscopy. While super resolved 3 color imaging exhibits already a great improvement, multicolor approaches with more colors might also be feasible in sequential approaches using DNA-conjugated antibodies (Chang et al., 2017; Schueder et al., 2017). A limitation of our approach might be the labeling density of cristae as high expansion factors of ten or even twenty will dilute the signal 1000- or 8000-fold, which results in insufficient signal to noise. Here, signal amplification methods might improve this issue as recently published (Kishi et al., 2019). Moreover, not all dyes are suitable for ExM as especially cyanines suffer severely during the gelation procedure. For ExM our most preferred and reliable dyes are Alexa Fluor 488, CF 568, ATTO 643, and ATTO 647N. Of course, when using ExM, artifacts deriving from an anisotropic expansion or insufficient digestion always have to be considered. Hence, reference structures, like mitochondrial cristae, can serve to prove an isotropic expansion.

In our study, we successfully demonstrated the close proximity to cristae of Mic60/Mitofilin, a protein localized to cristae junction and essential for cristae formation (John et al., 2005; Hessenberger et al., 2017), and for the first time TFAM, a mitochondrial transcription factor located in the mitochondrial matrix and associated with mtDNA nucleoids. In comparison to the localization relative to cristae of TFAM and another mitochondrial matrix protein PRX3 a clear difference can be observed. While TFAM co-localizes with cristae, PRX3 shows an alternating signal (Figures 2, 3). 3-color imaging (Figure 4) and ExM in combination with SIM (Figures 3, 4g–j and Supplementary Figure S3) indicated the localization of TFAM to cristae junction, which would explain the observed changes in mtDNA nucleoids upon Mic60/Mitofilin depletion (Li et al., 2016), and would also be in agreement with the study of Yang et al. showing interaction between Mic60/Mitofilin and TFAM (Yang et al., 2015), as well as with our unpublished data, which show a strong reduction in the quantities of mitochondrial genome maintenance exonuclease 1 (MGME1/C20orf72) upon Mic60/Mitofilin knockdown. MGME1 is an exo-/endonuclease involved in the maintenance of proper 7S DNA levels in mitochondria and mtDNA repair (Kornblum et al., 2013; Szczesny et al., 2013). It is therefore possible that the changes in mitochondrial morphology are sensed and responded to through the positioning of nucleoids at cristae junction and the functional connection of some of the mtDNA-associated proteins with the MICOS complex. However, more evidence needs to be obtained to understand this connection, for which ExM of mitochondria might prove to be a useful tool.

Finally, we used ExM of mitochondria with MtCK-GFP labeled cristae to visualize the defects in cristae morphology after treatment with CCCP (Figure 5 and Supplementary Figure S4), which dissipates membrane potential and leads to the swelling of mitochondria and loss of cristae structure. With increasing length of incubation, we could observe successfully the loss of cristae integrity, which is in accordance with previous studies. Using our tool, we could also observe the loss of cristae after knockdown of Sam50 as reported before (Ott et al., 2015) further demonstrating the usefulness of the MtCK-GFP as a marker for investigating cristae morphology. The combination of cristae labeling and ExM we present here is therefore a useful and relatively simple method for monitoring changes in cristae structure upon various stimuli, which could include up- or downregulation of proteins, chemical treatment or infection. Also, this tool can be used for determining submitochondrial localization of novel proteins in regard to cristae, enabling simultaneous immunodecoration for multiple markers and requiring only the usage of widely available fluorescent confocal microscopy.

## Data Availability Statement

The raw data supporting the conclusions of this article will be made available by the authors, without undue reservation, to any qualified researcher.

## Author Contributions

TK, RG, MS, and VK-P conceived the study, wrote the manuscript, and edited the manuscript. TK and RG performed the experiments and analyzed the data. SG performed the cloning of the construct. VK-P and MS supervised the study. All authors contributed to the article and approved the submitted version.

## Conflict of Interest

The authors declare that the research was conducted in the absence of any commercial or financial relationships that could be construed as a potential conflict of interest.
