# Supplementary material for: Using Expansion Microscopy to Visualize and Characterize the Morphology of Mitochondrial Cristae
Source: Front Cell Dev Biol. 2020 Jul 15;8:617. doi: 10.3389/fcell.2020.00617 (PMC7373753; doi:10.3389/fcell.2020.00617)
Supplement: Supplementary file 1 [file Image_1.pdf]

Supporting Material

Using Expansion Microscopy to visualize and characterize the morphology of mitochondrial cristae

Tobias C. Kunz [1a], Ralph Götz [2a], Shiqiang Gao [3], Markus Sauer [2], Vera Kozjak-Pavlovic[1,*]

[1]Department of Microbiology, Julius-Maximilians-Universität Würzburg, Würzburg, Germany

[2]Department of Biotechnology and Biophysics, Julius-Maximilians-Universität Würzburg, Würzburg, Germany

[3]Department of Botany 1, Julius-Maximilans-Universität Würzburg, Würzburg, Germany

[a] These authors contributed equally to this work: Tobias C. Kunz and Ralph Götz

*Corresponding author: Vera Kozjak-Pavlovic, Chair of Microbiology, University of Würzburg, AM Hubland, D-97074 Würzburg, Germany, E-mail: vera.kozjak@uni-wuerzburg.de

Keywords: Expansion microscopy, mitochondria, cristae, fluorescence microscopy, super-resolution microscopy

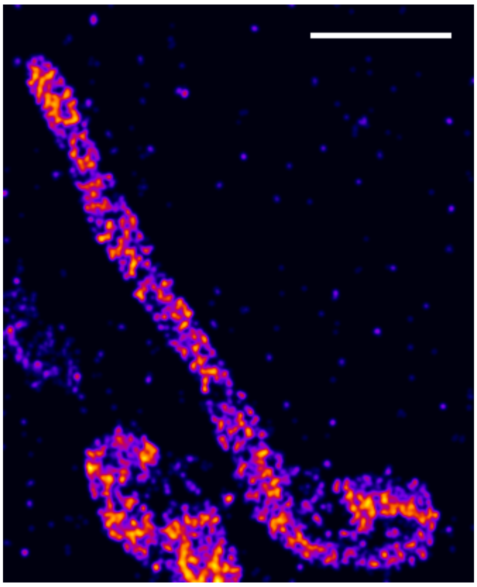

Supplemental Figure S1: dSTORM image of HeLa229 cells, transfected with MtCK-GFP for 24 hours, fixed and immunolabeled for GFP. Scale bar, 2 µm.

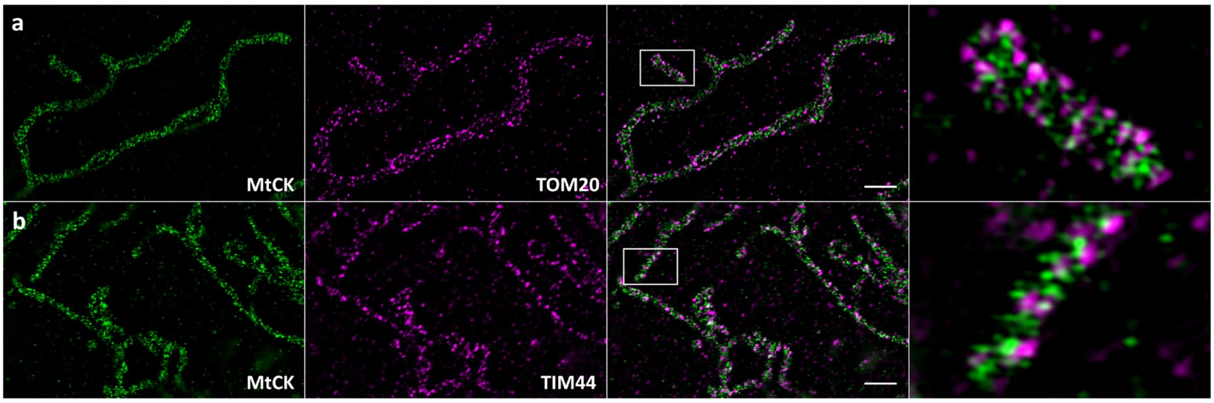

Supplemental Figure S2: 4x SIM-ExM of MtCK and mitochondrial marker. HeLa229 cells were transfected with MtCK-GFP for 24 hours, fixed, immunolabeled for GFP and (a) TOM20 or (b) TIM44 (magenta) and expanded. Within the overlay the zoomed region is indicated. Scale bars, 2 µm.

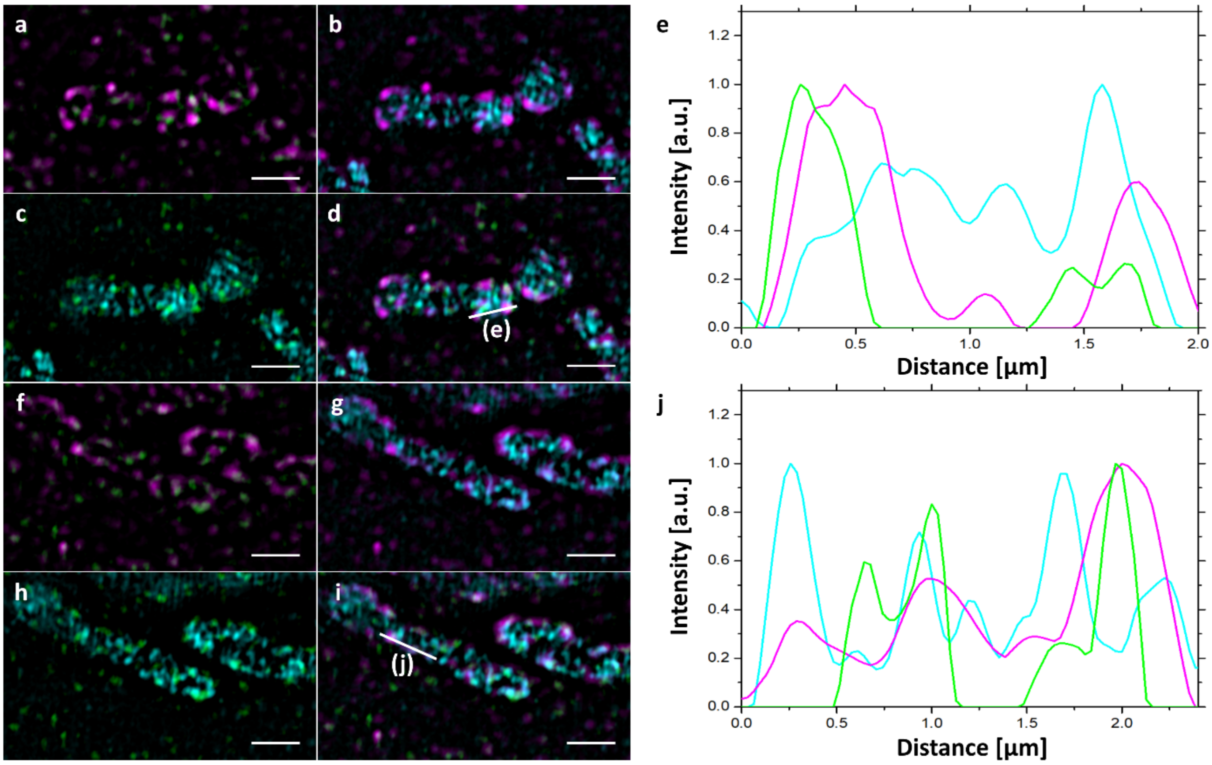

Supplemental Figure S3: 4x ExM of 3-color and SIM (a-d; f-i) images of the localization of mitochondrial proteins relative to mitochondrial cristae. Hela229 cells were transfected with MtCK-GFP for 24 hours, fixed, immunolabeled for GFP, Mitofilin and TFAM and expanded. (a, f) Mitofilin (magenta) and TFAM (green), (b, g) MtCK (cyan) and Mitofilin (magenta), (c, h) MtCK (cyan) and TFAM (green), (d, i) merge, with line indicating plot profiling shown in (e, j). Scale bars, 2 µm.

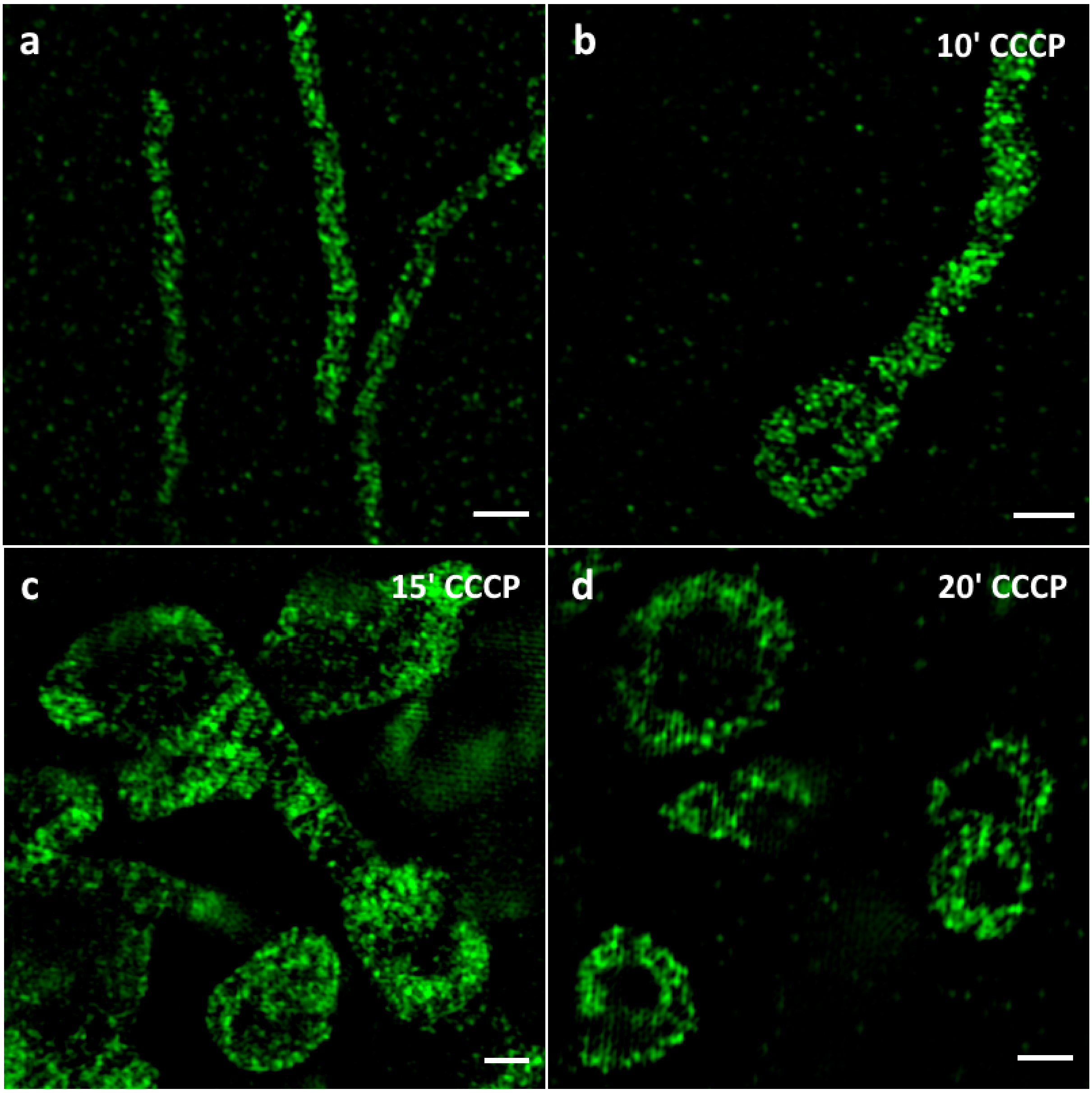

Supplemental Figure S4: SIM images of 4x expanded HeLa229 cells (a-d), transfected with MtCK-GFP (green), treated with 1 µM CCCP for 0 (a), 10 (b), 15 (c) or 20 (d) minutes. (e). Scale bars, 2 µm.
